# The Clinical Characteristic and Prognostic Value of Frailty in Elderly COPD Patients

**DOI:** 10.1002/jcsm.70381

**Published:** 2026-09-24

**Authors:** Tao Li, Ping Zhang, Qing Song, Cong Liu, Ling Lin, Yuqin Zeng, Ping Chen

**Affiliations:** ^1^ Department of Pulmonary and Critical Care Medicine The Second Xiangya Hospital, Central South University Changsha Hunan China; ^2^ Research Unit of Respiratory Disease Central South University Changsha Hunan China; ^3^ Clinical Medical Research Center for Pulmonary and Critical Care Medicine in Hunan Province China; ^4^ Diagnosis and Treatment Center of Respiratory Disease Central South University Changsha Hunan China

**Keywords:** COPD, dyspnoea, exacerbation, frailty

## Abstract

**Background and Objective:**

Frailty, as one of the extrapulmonary comorbidities of chronic obstructive pulmonary disease (COPD), is highly prevalent in COPD patients. The primary objective of this research was to assess how frailty influences subsequent exacerbation and augments the predictive capacity of dyspnoea or the exacerbation history among elderly COPD patients.

**Methods:**

The elderly COPD patients in this study were registered in the RealDTC study from June 2023 to March 2024. The Fried frailty phenotype was used to evaluate frailty, and patients were classified as robust, pre‐frail or frail. A Modified Medical Research Council Dyspnea Scale (mMRC) score of ≥ 2 was defined as more dyspnoea. For a risk index involved with frailty, dyspnoea and the exacerbation history, different scores were assigned to each factor. After 1 year of follow‐up, moderate to severe exacerbations and all‐cause mortality were recorded.

**Results:**

In total, 466 elderly patients were enrolled. Their mean age was 71.2 ± 4.5 years, with 89.0% male and 79.0% smokers. The mean COPD Assessment Test (CAT) score and forced expiratory volume in 1 s (FEV_1_)/forced vital capacity (FVC) were 14.1 ± 6.5 and 50.2 ± 11.6, respectively. Among all patients, 255 (54.7%) were in pre‐frailty and 132 (28.3%) were in frailty. Age (odds ratio [OR] = 1.135, 95% confidence interval [95%CI] = 1.060–1.216) and dyspnoea (OR = 3.654, 95%CI = 2.106–6.340) were correlated with pre‐frailty, and age (OR = 1.269, 95%CI = 1.120–1.438), dyspnoea (OR = 37.959, 95%CI = 12.694–113.503), educational level (OR = 0.303, 95%CI = 0.111–0.829), FEV_1_% predicted (OR = 0.976, 95%CI = 0.955–0.998) and BMI (OR = 0.845, 95%CI = 0.753–0.948) were independently associated with frailty in elderly COPD patients (all *p* < 0.05). The elderly COPD patients with pre‐frailty (OR = 2.182, 95% CI = 1.081–4.405) or frailty (OR = 3.377, 95%CI = 1.614–7.063) had an increased risk for future frequent exacerbation. Regarding all‐cause mortality, frailty has less effect on it in the 1‐year follow‐up of elderly COPD patients. By the ROC curve, frailty has the capacity (area under curve [AUC] = 0.607) to predict future exacerbation in elderly COPD patients. The combination of frailty and dyspnoea or the exacerbation history exhibited better predictive abilities than dyspnoea or the exacerbation history alone for future exacerbation, respectively (all *p* < 0.05).

**Conclusion:**

Frail elderly COPD patients present older age, lower BMI, poorer educational level, heavier symptom burden and worse pulmonary function. As a key COPD comorbidity, frailty increases the risk of future exacerbation, and combining it with dyspnoea or exacerbation history improves the predictive value for subsequent exacerbation.

**Abbreviations**
95% CI: 95% confidence interval
AUC: area under curve
BMI: body mass index
CAT: COPD Assessment Test
COPD: chronic obstructive pulmonary disease
FEV_1_
: forced expiratory volume in 1 s
FVC: forced vital capacity
GOLD: Global Initiative for Chronic Obstructive Lung Disease
HR: hazard ratio
ICS: inhaled corticosteroids
IQR: interquartile range
LABA: long‐acting β‐2 agonist
LAMA: long‐acting muscarinic antagonist
mMRC: Modified Medical Research Council Dyspnea Scale
OR: odds ratio
ROC: receiver operating characteristic
SD: standard deviation

## 1 Introduction

Chronic obstructive pulmonary disease (COPD) is a common chronic respiratory disease that affects many people's health. It has become the third leading cause of mortality worldwide [1]. COPD is now recognized as a systemic disease with several extrapulmonary comorbidities, including cardiovascular diseases, sarcopenia and frailty [2, 3]. Given the complexity and heterogeneity of COPD, it is difficult to assess it using a single index.

Frailty is described as an age‐related clinical syndrome characterized by decreased reserves across multiple physiological systems, which leads to marked vulnerability [4]. Previous work has shown that 10.7% of older adults in the community were living with frailty [5] and that frailty could substantially increase mortality risk in people aged over 65 years [6]. In chronic kidney disease, sarcopenia was demonstrated to be correlated with a high frailty index [4]. In China, people with possible sarcopenia or with sarcopenia exhibited a higher risk of frailty and, in longitudinal periods, a higher frailty index [7]. Cachexia has criteria overlapping with the Fried frailty phenotype [8, 9], and the occurrence of sarcopenia, cachexia or malnutrition could further increase the risk of all‐cause mortality in frail adults [10].

Research has demonstrated that COPD patients were at high risk for frailty [11]. COPD patients with frailty tend to have lower levels of physical activity and higher COPD Assessment Test (CAT) and Modified Medical Research Council Dyspnea Scale (mMRC) scores [12, 13]. Ushida et al. found that frailty was an independent risk factor for prolonged hospital stays in COPD patients [14]. These findings suggest a strong association between frailty and COPD and highlight the negative impact of frailty on people with COPD.

Global Initiative for Chronic Obstructive Lung Disease (GOLD), as the most vital reference for COPD, recommends symptom evaluations (mMRC and CAT) and the exacerbation history for COPD patient grouping which is mainly used for COPD treatment [15]. The mMRC is widely used to evaluate dyspnoea in COPD population, and dyspnoea is the most common complaint among COPD patients [16]. Several studies have shown that the mMRC was an independent factor for future exacerbations and mortality [17, 18]. Though it's very simple for COPD patients to complete mMRC due to its only one question, just dyspnoea could not provide enough information for prognosis of COPD. The exacerbation history has been reported as the best single predictor for future exacerbation in COPD [19]. The occurrence of an exacerbation increased the risk of subsequent exacerbation and mortality [20], which highlights the importance of preventing exacerbation in people with COPD. One study noted the potential bias in the exacerbation history because patients may misjudge their exacerbations [21]. Apart from the above single factors, the BODE index (BMI, airflow obstruction, dyspnoea, and exercise capacity) is a well‐established composite factor for prediction of long‐term mortality [22], and has been demonstrated to have the capacity of predicting severe exacerbation [23]. Exercise capacity of BODE index is assessed by 6‐min walk test, but the demand for long time and large space of 6‐min walk test requires more willingness of cooperation from the patient, which limits its utility at the outpatient department. A simple and practical composite index should be explored in COPD.

In this study, the primary aim was to examine the effect of frailty on future exacerbation and on enhancing the predictive abilities of dyspnoea or the exacerbation history for future exacerbation in elderly COPD patients. The secondary aim was to explore the factors associated with pre‐frailty and frailty in this population.

## 2 Methods

### 2.1 Study Participants and Design

This was a single‐centre, prospective study, and participants were drawn from the RealDTC (ChiCTR‐POC‐17010431) study between June 2023 and March 2024 [24]. According to the GOLD 2023 document, COPD was diagnosed when the post‐bronchodilator ratio of forced expiratory volume in 1 s to forced vital capacity (FEV_1_/FVC) was < 70%. Patients aged ≥ 65 years were enrolled, excluding those with lung cancer, previous lung surgery, or mental disorders. The study was approved by the Ethics Committee of the Second Xiangya Hospital (2016076). Written informed consent was obtained from all patients.

### 2.2 Data Collection

Baseline data included age, gender, educational level, height, weight, CAT, mMRC, frailty, pulmonary function, smoking pack‐years, occupational exposure, biofuel exposure, exacerbation history, inhaled treatment, and comorbidities.

Smokers were defined as those who had smoked more than 10 pack‐years in their lifetime; others were classified as non‐smokers [25]. Occupational exposure referred to exposure to dust, metals, chemical substances, or other environmental agents for at least 1 year and at least 8 h per day [26]. Biofuel exposure referred to the use of biomass fuels (wood, grass, charcoal, crop residues) for cooking or heating for at least 1 year and for at least 2 h per day [27]. Comorbidities included asthma, bronchiectasis, pulmonary tuberculosis, cardiovascular diseases, and diabetes. Cardiovascular diseases included cardiomyopathy, coronary heart disease, hypertension, heart valve disease, arrhythmia, and related conditions. Symptom questionnaires, including CAT and mMRC, were self‐administered; if needed, trained staff explained specific items to patients.

After 1 year of follow‐up, we collected the number of moderate and severe exacerbations and recorded mortality. All patients continued their inhaled treatment without changes during the follow‐up period.

### 2.3 Variable Definition

In this study, an mMRC of ≥ 2 was defined as more dyspnoea, and an mMRC of < 2 was defined as less dyspnoea. The exacerbation history referred to patients with one or more moderate or severe exacerbations in the past year. An exacerbation was defined as an acute worsening of respiratory symptoms requiring additional treatment, including corticosteroids and/or antibiotics (moderate exacerbation) or hospitalization (severe exacerbation) [28]. Frequent exacerbations were defined as at least two exacerbations per year [29]. Hospitalization referred to admission due to a COPD exacerbation. All‐cause mortality referred to death from any disease or accident during the follow‐up period.

According to the GOLD 2026 document, patients were classified into three groups [15]:

- Group A: CAT score < 10, and mMRC score of 0–1
- Group B: CAT score of ≥10 or mMRC score of ≥ 2
- Group E: ≥1 exacerbation per year

GOLD stages were defined as follows:

- Stage 1: FEV_1_% predicted ≥ 80%
- Stage 2: 50% ≤ FEV_1_% predicted < 80%
- Stage 3: 30% ≤ FEV_1_% predicted < 50%
- Stage 4: FEV_1_% predicted < 30%

### 2.4 Frailty Definition

Frailty in this study was defined by Fried frailty phenotype (FFP) [9]. he FFP includes five components: weight loss, exhaustion, physical activity, walk time, and grip strength.

1 Weight loss: In the past year, has the patient lost more than 5 kg or more than 5% of their body weight unintentionally (not due to dieting or exercise)?
2 Exhaustion: “How often in the past week did you feel this way?”

- I felt that everything I did was an effort.
- I could not get going.

Scores: 0 = rarely or none of the time (< 1 day), 1 = some or a little of the time (1–2 days), 2 = a moderate amount of time (3–4 days), 3 = most of the time (> 4 days). A score of 2 or 3 on either item indicates exhaustion.

3 Physical activity: Low physical activity was defined as < 383 kcal per week for male and < 270 kcal per week for female. For male, a weekly consumption of 383 kcal equals walking for at least 2.5 h per week. For female, a weekly consumption of 270 kcal equals walking for at least 2 h per week. Activities included walking, biking, chores, hiking, jogging, and similar forms of movement.
4 Walk time: Time to walk 15 ft

- Male: height ≤ 173 cm, ≥7 s; height > 173 cm, ≥6 s
- Female: height ≤ 159 cm, ≥7 s; height > 159 cm, ≥6 s

5 Grip strength: Assessment of upper limb strength

Male:

- Body mass index (BMI) ≤ 24.0 kg/m^2^, grip strength ≤ 29 kg
- BMI 24.1–28.0 kg/m^2^, grip strength ≤ 30 kg
- BMI > 28.0 kg/m^2^, grip strength ≤ 32 kg

Female:

- BMI ≤ 23.0 kg/m^2^, grip strength ≤ 17 kg
- BMI 23.1–26.0 kg/m^2^, grip strength ≤17.3 kg
- BMI 26.1–29.0 kg/m^2^, grip strength ≤ 18 kg
- BMI > 29.0 kg/m^2^, grip strength ≤ 21 kg

The elderly COPD patients meeting three or more FFP components were classified as frailty; those meeting one or two components were classified as pre‐frailty; and those meeting none were classified as robust. At the beginning of the assessment, our dedicated trained staff will introduce the components included in FFP. For items related to weight loss, exhaustion and physical activity, our staff will explain the implications of each question, and patients will complete these questionnaires independently. In terms of walking time and grip strength assessments, our staff will provide standardized instructions on how to conduct the assessments and record the relevant data accordingly.

As for risk index defined by this study, scores were assigned as follows: robust = 0, pre‐frailty = 1, frailty = 2; less dyspnoea = 0, more dyspnoea = 1, no exacerbation history = 0 and one or more exacerbation histories = 1. The Risk Index 1 is the combination of frailty and dyspnoea, and Risk Index 2 is the combination of frailty and the exacerbation history.

### 2.5 Data Analysis

SPSS 26.0 (IBM, Armonk, NY, USA) was used for statistical analysis. Continuous variables are presented as mean ± standard deviation or as median with interquartile range. Categorical variables are presented as number (percentage). Differences between robust group and pre‐frailty or frailty group were examined using the Mann–Whitney U test for continuous variables and the χ^2^ test for categorical variables. Variables that showed significant differences in comparison between robust group and pre‐frailty or frailty group were entered into the subsequent logistic regression. Logistic regression was used to identify factors associated with pre‐frailty or frailty.

The χ^2^ test was used to compare rates of future exacerbation, frequent exacerbation, hospitalization, and all‐cause mortality between the robust group and the pre‐frailty or frailty group. Logistic regression was used to analyse whether frailty, dyspnoea, the exacerbation history, and risk index were associated with future exacerbation, frequent exacerbation, and hospitalization, adjusted for age, gender, BMI, FEV_1_% predicted, smoking status, occupational exposure, biofuel exposure, education level, inhaled treatment, and comorbidities. Receiver operating characteristic (ROC) curves were used to assess the predictive value of frailty, dyspnoea, the exacerbation history, and risk index. The DeLong test was used to compare ROC curves across these predictors. The Hosmer–Lemeshow test was used to assess the goodness‐of‐fit of all logistic regression models.

## 3 Results

### 3.1 Baseline Data and Comparison Among Robust, Pre‐Frailty and Frailty Groups in Elderly COPD Patients

In total, 466 elderly COPD patients were included. Their mean age was 71.2 ± 4.5 years, with 88.6% being male and 79.0% being smokers. The mean CAT score was 14.1 ± 6.5, and the median mMRC score was 2. The mean FEV_1_% predicted and FEV_1_/FVC were 57.7% ± 21.3 and 50.2 ± 11.6, respectively (Table 1).

**TABLE 1:** The characteristics of elderly COPD patients at baseline.

Characteristics	Total (*N* = 466)
**Age, mean ± SD**	71.2 ± 4.5
**Gender, number (%)**	
**Male**	413 (88.6)
**Female**	53 (11.4)
**Smoking pack‐year, median (IQR)**	45 (19.6, 57)
**BMI, mean ± SD**	22.9 ± 3.5
**CAT, mean ± SD**	14.1 ± 6.5
**mMRC, median (IQR)**	2 (1, 2)
**FEV**_**1**_ **% predicted, (Mean ± SD)**	57.7 ± 21.3
**FEV**_**1**_ **/FVC, (Mean ± SD)**	50.2 ± 11.6
**Educational level, number (%)**	
**Junior school and below**	366 (78.5)
**High school and above**	100 (21.5)
**Smoking status, number (%)**	
**Non‐smoker**	98 (21.0)
**Smoker**	368 (79.0)
**Occupational exposure, number (%)**	
**No**	281 (60.3)
**Yes**	185 (39.7)
**Biofuel exposure, number (%)**	
**No**	328 (70.4)
**Yes**	138 (29.6)
**The exacerbation history, number (%)**	
**No**	169 (36.3)
**Yes**	297 (63.7)
**GOLD 2026, number (%)**	
**A**	48 (10.3)
**B**	121 (26.0)
**E**	297 (63.7)
**GOLD stage, number (%)**	
**1**	75 (16.1)
**2**	194 (41.6)
**3**	158 (33.9)
**4**	39 (8.4)
**Inhaled treatment, number (%)**	
**LAMA**	24 (5.2)
**LABA/ICS**	142 (30.5)
**LABA/LAMA**	29 (6.2)
**LABA/LAMA/ICS**	260 (55.8)
**Others**	11 (2.3)
**Weight loss, number (%)**	
**No**	400 (85.8)
**Yes**	66 (14.2)
**Physical activity, number (%)**	
**No**	121 (26.0)
**Yes**	345 (74.0)
**Exhaustion, median (IQR)**	0 (0, 3)
**Walk time, s, mean ± SD**	6.9 ± 2.2
**Grip strength, kg, mean ± SD**	25.6 ± 7.6
**Comorbidity, number (%)**	
**Asthma**	100 (21.5)
**Bronchiectasis**	21 (4.5)
**Pulmonary tuberculosis**	10 (2.1)
**Cardiovascular diseases**	125 (26.8)
**Diabetes**	27 (5.8)

*Note:* Others included SAMA, SABA, and no inhalation therapy. Cardiovascular diseases include cardiomyopathy, coronary heart disease, hypertension, heart valve disease, arrhythmia and others.

Abbreviations: COPD, chronic obstructive pulmonary disease; BMI, Body Mass Index; CAT, COPD Assessment Test; mMRC, Modified Medical Research Council Dyspnea Scale; FEV_1_, forced expiratory volume in 1 s; FVC, forced vital capacity; ICS, inhaled corticosteroids; LABA, long‐acting β‐2‐agonist; LAMA, long‐acting muscarinic antagonist; SABA, short‐acting β2‐agonist, SAMA, short‐acting muscarinic agonist, GOLD, Global Initiative for Chronic Obstructive Lung Disease; SD, standard deviation; IQR, interquartile range.

Based on the FFP, 54.7% of participants were in pre‐frailty and 28.3% were in frailty (Table 2). Compared with robust group, pre‐frailty group had higher age, lower FEV_1_% predicted, a greater proportion with lower education level, and higher CAT and mMRC scores. In addition to these variables, frailty group also had a lower BMI and FEV_1_/FVC and a greater proportion of patients with the exacerbation history, in GOLD group E, in GOLD stage 4, and receiving LABA/LAMA/ICS treatment (all *p* < 0.05) (Table 2).

**TABLE 2:** The comparison of clinical characteristics among robust, pre‐frailty and frailty groups.

Characteristics	Robust	Pre‐frailty	Frailty	*p* ^a^	*p* ^b^
**Number**	79 (17.0)	255 (54.7)	132 (28.3)		
**Age, mean ± SD**	69.5 ± 3.6	71.5 ± 4.6	71.8 ± 4.8	< 0.001	< 0.001
**Gender, number (%)**				0.562	0.948
**Male**	71 (89.9)	223 (87.5)	119 (90.2)		
**Female**	8 (10.1)	32 (12.5)	13 (9.8)		
**Smoking pack‐year, median (IQR)**	44 (15, 53)	43 (16.5, 59)	47 (25, 57.5)	0.738	0.209
**BMI, mean ± SD**	23.3 ± 2.8	23.3 ± 3.3	21.9 ± 3.9	0.904	< 0.001
**CAT, mean ± SD**	10.7 ± 5.2	12.6 ± 5.5	18.8 ± 6.6	0.003	< 0.001
**mMRC, median (IQR)**	1 (1, 2)	2 (1, 2)	3 (2, 3)	< 0.001	< 0.001
**Dyspnea, number (%)**				< 0.001	< 0.001
**Less dyspnoea**	54 (68.4)	99 (38.8)	8 (6.1)		
**More dyspnoea**	25 (31.6)	156 (61.2)	124 (93.9)		
**FEV**_**1**_ **% predicted, mean ± SD**	66.0 ± 19.9	60.2 ± 20.6	48.1 ± 20.0	0.024	< 0.001
**FEV**_**1**_ **/FVC, mean ± SD**	53.4 ± 10.2	51.4 ± 11.5	45.8 ± 11.5	0.235	< 0.001
**Educational level, number (%)**				0.034	< 0.001
**Junior school and below**	52 (65.8)	198 (77.6)	116 (87.9)		
**High school and above**	27 (34.2)	57 (22.4)	16 (12.1)		
**Smoking status, number (%)**				0.876	0.553
**Non‐smoker**	17 (21.5)	57 (22.4)	24 (18.2)		
**Smoker**	62 (78.5)	198 (77.6)	108 (81.8)		
**Occupational exposure, number (%)**				0.545	0.528
**No**	45 (57.0)	155 (60.8)	81 (61.4)		
**Yes**	34 (43.0)	100 (39.2)	51 (38.6)		
**Biofuel exposure, number (%)**				0.225	0.104
**No**	61 (77.2)	179 (70.2)	88 (66.7)		
**Yes**	18 (22.8)	76 (29.8)	44 (33.3)		
**The exacerbation history, number (%)**				0.181	< 0.001
**No**	38 (48.1)	101 (39.6)	30 (22.7)		
**Yes**	41 (51.9)	154 (60.4)	102 (77.3)		
**GOLD 2026, number (%)**				0.363	< 0.001
**A**	14 (17.7)	33 (12.9)	1 (0.8)		
**B**	24 (30.4)	68 (26.7)	29 (22.0)		
**E**	41 (51.9)	154 (60.4)	102 (77.2)		
**GOLD stage, number (%)**				0.074	< 0.001
**1**	21 (26.6)	45 (17.6)	9 (6.8)		
**2**	36 (45.6)	115 (45.1)	43 (32.6)		
**3**	22 (27.8)	83 (32.6)	53 (40.2)		
**4**	0 (0.0)	12 (4.7)	27 (20.4)		
**Inhaled treatment, number (%)**				0.227	0.008
**LAMA**	8 (10.1)	14 (5.5)	2 (1.5)		
**LABA/ICS**	9 (11.4)	15 (5.9)	5 (3.8)		
**LABA/LAMA**	23 (29.1)	77 (30.2)	42 (31.8)		
**LABA/LAMA/ICS**	38 (48.1)	143 (56.1)	79 (59.9)		
**Others**	1 (1.3)	6 (2.3)	4 (3.0)		
**Weight loss, number (%)**				< 0.001	< 0.001
**No**	79 (100.0)	225 (88.2)	96 (72.7)		
**Yes**	0 (0.0)	30 (11.8)	36 (27.3)		
**Physical activity, number (%)**				0.001	< 0.001
**No**	0 (0.0)	31 (12.2)	90 (68.2)		
**Yes**	79 (100.0)	224 (87.8)	42 (31.8)		
**Exhaustion, median (IQR)**	0 (0, 0)	0 (0, 0)	3 (3, 6)	< 0.001	< 0.001
**Walk time, s, mean ± SD)**	5.5 ± 0.8	6.7 ± 2.0	8.3 ± 2.3	< 0.001	< 0.001
**Grip strength, kg, mean ± SD**	33.1 ± 5.4	25.6 ± 6.9	21.1 ± 6.5	< 0.001	< 0.001
**Comorbidity, number (%)**					
**Asthma**	21 (26.6)	53 (20.8)	26 (19.7)	0.278	0.245
**Bronchiectasis**	4 (5.1)	13 (5.1)	4 (3.0)	1.000	0.476
**Pulmonary tuberculosis**	1 (1.3)	7 (2.7)	2 (1.5)	0.686	1.000
**Cardiovascular diseases**	21 (26.6)	68 (26.7)	36 (27.2)	0.988	0.913
**Diabetes**	4 (5.1)	15 (5.9)	8 (6.1)	1.000	1.000

*Note:* Others include SAMA, SABA and no inhalation therapy. Cardiovascular diseases include cardiomyopathy, coronary heart disease, hypertension, heart valve disease, arrhythmia and others.

Abbreviations: COPD, chronic obstructive pulmonary disease; BMI, Body Mass Index; CAT, COPD Assessment Test; mMRC, Modified Medical Research Council Dyspnea Scale; FEV_1_, forced expiratory volume in 1 s; FVC, forced vital capacity; ICS, inhaled corticosteroids; LABA, long‐acting β‐2‐agonist; LAMA, long‐acting muscarinic antagonist; SABA, short‐acting β2‐agonist, SAMA, short‐acting muscarinic agonist, GOLD, Global Initiative for Chronic Obstructive Lung Disease; SD, standard deviation; IQR, interquartile range.

^a^ The comparison between the pre‐frailty group and the robust group.

^b^ The comparison between the frailty group and the robust group.

### 3.2 Relative Factors for Pre‐Frailty and Frailty in Elderly COPD Patients

Logistic regression showed that age (odds ratio [OR] = 1.136, 95% confidence interval [CI] = 1.063–1.215) and dyspnoea (OR = 3.654, 95% CI = 2.106–6.340) were associated with pre‐frailty (all *p* < 0.05) (Table 3).

**TABLE 3:** The relative factors for pre‐frailty and frailty in the elderly COPD patients by logistic regression analysis.

Factors	OR (95%CI)	*p*
**Pre‐frailty** a		
**Age**	1.135 (1.060–1.216)	< 0.001
**Dyspnea**		
**Less dyspnoea**	Reference	
**More dyspnoea**	3.654 (2.106–6.340)	< 0.001
**Frailty** b		
**Age**	1.269 (1.120–1.438)	< 0.001
**Educational level**		
**Junior school and below**	Reference	
**High school and above**	0.303 (0.111–0.829)	0.020
**FEV**_**1**_ **% predicted**	0.976 (0.955–0.998)	0.032
**BMI**	0.845 (0.753–0.948)	0.004
**Dyspnea**		
**Less dyspnoea**	Reference	
**More dyspnoea**	37.959 (12.694–113.503)	< 0.001

*Note:* Model 1 was adjusted for age, FEV_1_% predicted, dyspnoea and educational level. Model 2 was adjusted for age, BMI, FEV1% predicted, educational level, dyspnoea, the exacerbation history. The *p* value of the Hosmer–Lemeshow test for Models 1 and 2 > 0.05. Pre‐frailty and frailty are different outcomes for patients, which means that they are as two different dependent variables included in logistic regression, respectively.

^a^ Model 1 for pre‐frailty.

^b^ Model 2 for frailty.

Age (OR = 1.269, 95% CI = 1.120–1.438), education level (OR = 0.303, 95% CI = 0.111–0.829), FEV_1_% predicted (OR = 0.976, 95% CI = 0.955–0.998), BMI (OR = 0.845, 95% CI = 0.753–0.948), and dyspnoea (OR = 37.959, 95% CI = 12.694–113.503) were associated with frailty (all *p* < 0.05) (Table 3).

### 3.3 Effect of Pre‐Frailty and Frailty for Future Exacerbation and All‐Cause Mortality in Elderly COPD Patients

After adjustment for confounding factors, pre‐frailty (OR = 2.182, 95% CI = 1.081–4.405) and frailty (OR = 3.377, 95% CI = 1.614–7.063) were independent risk factors for future frequent exacerbation (*p* < 0.05). There were no significant differences in 1‐year all‐cause mortality between robust and pre‐frailty or frailty groups (Tables 4 and 5).

**TABLE 4:** The rates of future exacerbation, frequent exacerbation, hospitalization and all‐cause mortality among robust, pre‐frailty and frailty groups in the elderly COPD patients.

Groups	Robust	Pre‐frailty	Frailty	*p* ^a^ value	*p* ^b^ value
**Exacerbation**	27 (34.2)	132 (51.8)	77 (58.3)	0.006	< 0.001
**Frequent exacerbation**	11 (13.9)	69 (27.1)	52 (39.4)	0.017	< 0.001
**Hospitalization**	14 (17.7)	73 (28.6)	51 (38.6)	0.054	0.001
**All‐cause mortality**	0 (0.0)	4 (1.6)	5 (3.8)	0.576	0.160

^a^ The pre‐frailty group was compared to the robust group.

^b^ The frailty group was compared to the robust group.

**TABLE 5:** The effect of frailty, dyspnoea and the exacerbation history for future exacerbation, frequent exacerbation and hospitalization in the elderly COPD patients by logistic regression analysis.

Factors	Any exacerbation^a^		Frequent exacerbation^b^		Hospitalization^c^	
	OR (95%CI)	*p*	OR (95%CI)	*p*	OR (95%CI)	*p*
**Robust**	Reference		Reference		Reference	
**Pre‐frailty**	1.731 (0.980–3.057)	0.058	2.182 (1.081–4.405)	0.029	1.359 (0.681–2.712)	0.384
**Frailty**	1.379 (0.703–2.705)	0.350	3.377 (1.614–7.063)	0.001	1.317 (0.605–2.866)	0.487
**Dyspnea**						
**Less dyspnoea**	Reference		Reference		Reference	
**More dyspnoea**	1.826 (1.212–2.750)	0.004	1.282 (0.757–2.170)	0.355	2.325 (1.430–3.782)	< 0.001
**The exacerbation history**						
**No**	Reference		Reference		Reference	
**Yes**	2.634 (1.749–3.967)	< 0.001	2.424 (1.500–3.915)	< 0.001	1.936 (1.209–3.100)	0.006

*Note:* The model 1, 2 and 3 was adjusted for age, gender, BMI, dyspnoea, FEV_1_% predicted, smoking status, occupational exposure, biofuel exposure, educational level, exacerbation history, inhaled treatment and comorbidities. The *p* value of Hosmer–Lemeshow test for Models 1, 2 and 3 was > 0.05. Any exacerbation, frequent exacerbation and hospitalization are different outcomes for patients, which mean that they are as three different dependent variables included in logistic regression, respectively. All models are adjusted by the same factors.

^a^ Model 1 for for any exacerbation.

^b^ Model 2 for for frequent exacerbation.

^c^ Model 2 for frailty.

### 3.4 Effect of Risk Index Combined With Frailty and Dyspnoea or the Exacerbation History for Future Exacerbation in Elderly COPD Patients

To further analyse the effect of frailty combined with dyspnoea or the exacerbation history, scores were assigned as follows: robust = 0, pre‐frailty = 1, frailty = 2; less dyspnoea = 0, more dyspnoea = 1, no exacerbation history = 0 and one or more exacerbation history = 1. The Risk Index 1 (the combination of frailty and dyspnoea) and Risk Index 2 (the combination of frailty and the exacerbation history) ranged from 0 to 3.

After adjustment, the Risk Index 1 of ≥ 1 was associated with a 2.1‐ to 3.7‐fold increased risk of future exacerbation and a 4.3‐ to 6.8‐fold increased risk of frequent exacerbation. The Risk Index 1 of ≥ 2 was associated with a 4.0‐ to 4.3‐fold increased risk of future hospitalization. The Risk Index 2 of ≥ 2 was correlated with a 2.6‐ to 4.1‐fold increased risk of future exacerbation and a 5.1‐ to 9.5‐fold increased risk of future frequent exacerbation (all *p* < 0.05) (Table 6).

**TABLE 6:** The risk value of Risk Indexes 1 and 2 for future exacerbation, frequent exacerbation and hospitalization in the elderly COPD patients.

Factors	Any exacerbation		Frequent exacerbation		Hospitalization	
	OR (95%CI)	*p*	OR (95%CI)	*p*	OR (95%CI)	*p*
**Risk Index 1**						
**0**	Reference		Reference		Reference	
**1**	2.161 (1.048–4.455)	0.037	4.372 (1.447–13.209)	0.009	2.407 (0.913–6.346)	0.076
**2**	3.717 (1.846–7.485)	< 0.001	4.722 (1.603–13.908)	0.005	4.324 (1.719–10.880)	0.002
**3**	2.740 (1.331–5.642)	0.006	6.888 (2.317–20.474)	< 0.001	4.038 (1.573–10.363)	0.004
**Risk Index 2**						
**0**	Reference		Reference		Reference	
**1**	1.892 (0.824–4.344)	0.133	2.739 (0.784–9.574)	0.115	1.202 (0.449–3.220)	0.714
**2**	3.622 (1.581–8.298)	0.002	5.104 (1.507–17.292)	0.009	1.810 (0.688–4.764)	0.230
**3**	4.122 (1.663–10.217)	0.002	9.583 (2.768–33.180)	< 0.001	2.474 (0.886–6.908)	0.084

*Note:* We defined that robust was 0, pre‐frailty was 1, frailty was 2, less dyspnoea was 0, more dyspnoea was 1, no exacerbation history was 0 and one or more exacerbation history was 1. Risk Index 1 was the combination of frailty and dyspnoea, and Risk Index 2 was the combination of frailty and the exacerbation history. The model of Risk Index 1 was adjusted for age, gender, BMI, FEV_1_% predicted, smoking status, occupational exposure, biofuel exposure, educational level, the exacerbation history, inhaled treatment and comorbidities. The model of Risk Index 2 was adjusted for age, gender, BMI, FEV1% predicted, dyspnoea, smoking status, occupational exposure, biofuel exposure, educational level, inhaled treatment and comorbidities. The *p* value of Hosmer‐Lemeshow test for all models was > 0.05.

### 3.5 ROC Curve Analysis of Frailty, Dyspnoea, the Exacerbation History, and Risk Index for Future Exacerbation in Elderly COPD Patients

**FIGURE 1:**
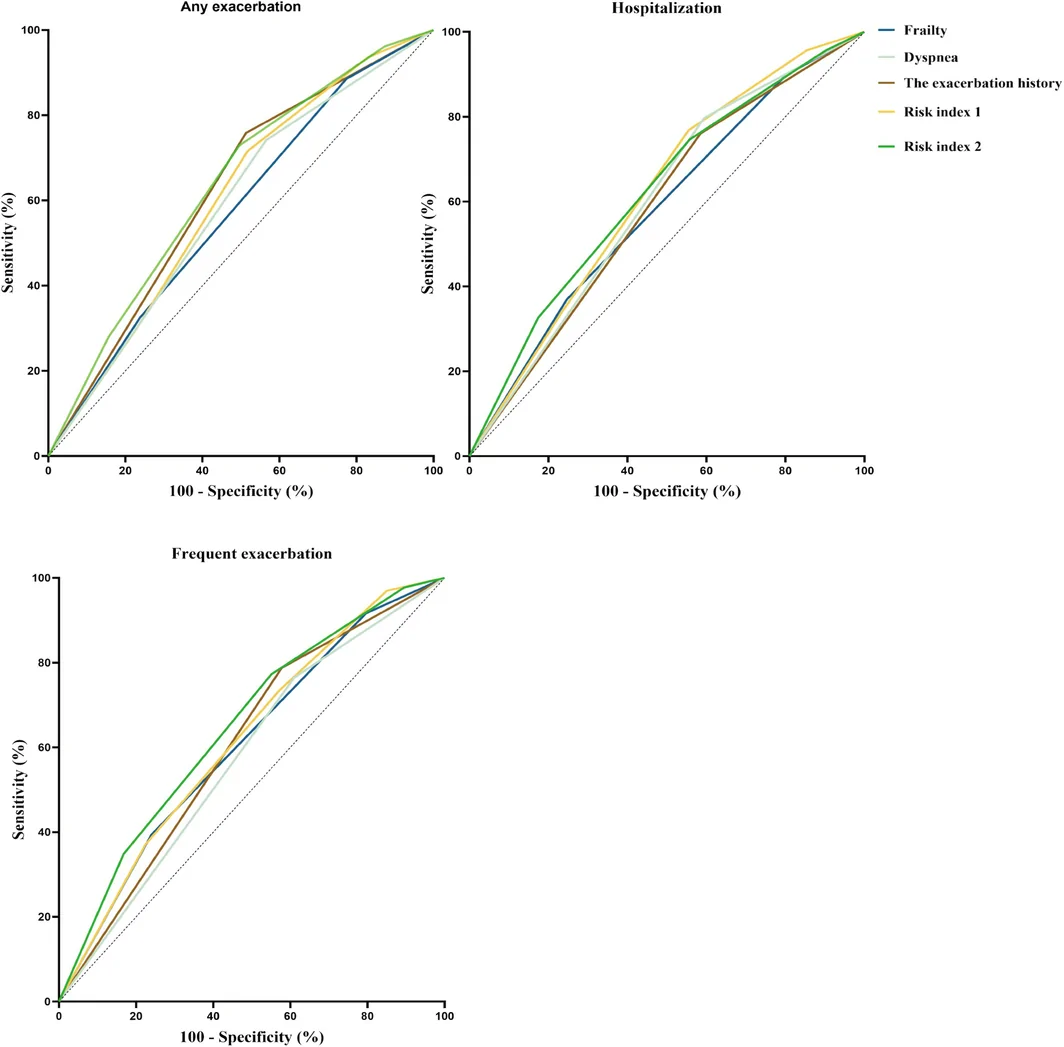
The ROC curve of frailty, dyspnoea, the exacerbation history and risk index for future exacerbation, frequent exacerbation and hospitalization.

All ROC curves and values of AUC of frailty, dyspnoea, the exacerbation history, and risk index for future exacerbation were in Figure 1 and Table 7. By Delong test, the predictive ability of frailty was similar to those of dyspnoea and the exacerbation history, and the predictive value was comparable between Risk Index 1 and the exacerbation history for future exacerbation (Table 8).

**TABLE 7:** The AUC of frailty, dyspnoea, the exacerbation history and risk index (1 and 2) for future exacerbation, frequent exacerbation and hospitalization by ROC curve.

Factors	Any exacerbation		Frequent exacerbation		Hospitalization	
	AUC (95%CI)	*p*	AUC (95%CI)	*p*	AUC (95%CI)	*p*
**Dyspnoea**	0.588 (0.536–0.640)	0.001	0.577 (0.521–0.633)	0.009	0.601 (0.547–0.656)	0.001
**The exacerbation history**	0.623 (0.572–0.674)	< 0.001	0.605 (0.550–0.660)	< 0.001	0.588 (0.533–0.643)	0.003
**Frailty**	0.576 (0.524–0.628)	0.004	0.607 (0.551–0.663)	< 0.001	0.586 (0.530–0.642)	0.004
**Risk Index 1**	0.603 (0.552–0.655)	< 0.001	0.619 (0.564–0.673)	< 0.001	0.618 (0.566–0.672)	< 0.001
**Risk Index 2**	0.636 (0.586–0.687)	< 0.001	0.649 (0.595–0.703)	< 0.001	0.623 (0.568–0.678)	< 0.001

*Note:* We defined that robust was 0, pre‐frailty was 1, frailty was 2, less dyspnoea was 0, more dyspnoea was 1, no exacerbation history was 0 and one or more exacerbation history was 1. Risk Index 1 was the combination of frailty and dyspnoea, and Risk Index 2 was the combination of frailty and the exacerbation history.

**TABLE 8:** The Delong test was used to compare the ability of ROC curve of frailty, dyspnoea, the exacerbation history and risk index (1 and 2) for future exacerbation, frequent exacerbation and hospitalization.

Compared Factors	Any exacerbation		Frequent exacerbation		Hospitalization	
	Difference of AUC	*p*	Difference of AUC	*p*	Difference of AUC	*p*
**Frailty vs dyspnoea**	−0.012	0.624	0.030	0.275	−0.016	0.567
**Frailty vs the exacerbation history**	−0.047	0.115	0.002	0.943	−0.002	0.943
**Risk Index 1 vs dyspnoea**	0.015	0.334	0.041	0.021	0.017	0.348
**Risk Index 1 vs frailty**	0.027	0.019	0.011	0.373	0.033	0.008
**Risk Index 1 vs the exacerbation history**	−0.020	0.518	0.013	0.665	0.030	0.315
**Risk Index 2 vs the exacerbation history**	0.014	0.472	0.044	0.031	0.035	0.077
**Risk Index 2 vs frailty**	0.060	< 0.001	0.042	0.004	0.038	0.010

*Note:* We defined that robust was 0, pre‐frailty was 1, frailty was 2, less dyspnoea was 0, more dyspnoea was 1, no exacerbation history was 0 and one or more exacerbation history was 1. Risk Index 1 was the combination of frailty and dyspnoea, and Risk Index 2 was the combination of frailty and the exacerbation history.

The Risk Index 1 presented a better value than dyspnoea for future frequent exacerbation and then frailty for future exacerbation and hospitalization. The Risk Index 2 exhibited a better value than the exacerbation history for future frequent exacerbation and then frailty for future exacerbation (all *p* < 0.05) (Table 8).

## 4 Discussion

This study stated that frailty was in high prevalence in elderly COPD patients and increased the risk of future frequent exacerbation of elderly COPD patients. Frailty, as a possible supplementary and multi‐dimensional evaluation, could boost the predictive value of either dyspnoea or the exacerbation history for future exacerbation.

In China, the prevalence of COPD among individuals aged over 60 years of age exceeds 27% [30]. Frailty was also highly prevalent in older adults and has a negative effect on mortality in people aged over 65 years [5, 6]. Based on these findings, this study focused on COPD patients aged over 65 years. The overall prevalence of frailty and pre‐frailty has been reported as 36% and 43% in COPD, respectively [31]. In this study, 28.3% of participants were in frailty and 54.7% were in pre‐frailty, which was consistent with previous work.

Several relative factors were identified for pre‐frailty and frailty in elderly COPD patients. Age and dyspnoea were associated with both pre‐frailty and frailty. Frailty was also associated with educational level, BMI, and FEV_1_% predicted. Frailty was considered an age‐related syndrome [4], so the association between age and frailty was expected. Frailty in COPD was proved to be correlated with mMRC [12], which is same as our finding. Dyspnoea may lead to reduced physical activity, which may further increase dyspnoea and create a vicious cycle. Our previous work showed that the lower education level increased the risk of all‐cause mortality in COPD patients [24], and other studies have also reported an association between low education level and frailty [32]. People with lower education levels may have less disease‐related knowledge, fewer resources to sustain treatment, and fewer opportunities to maintain a healthy lifestyle, which may explain the link between education and frailty. Further work is needed to explore the combined effects of educational level and frailty on the prognosis of COPD patients. A systematic review reported that both underweight and obesity increased the risk of frailty in older adults [33]. Among individuals with COPD, higher risks of exacerbation and mortality have been shown in those with a low rather than high BMI [34]. This suggests that the negative correlation between frailty and BMI may be a specific feature in elderly COPD patients. Reduced FEV_1_% predicted had been linked to a higher risk of frailty [35]. FEV_1_% predicted was widely used to assess COPD severity, and lower FEV_1_% predicted reflected more severe disease. This partly accounts for this correlation between frailty and FEV_1_% predicted.

To further analyse the effect of frailty in elderly COPD patients, this study collected 1‐year follow‐up data and found that frailty and pre‐frailty were independent factors for future frequent exacerbation. Scarlata et al. reported that COPD patients with the frequent exacerbation history had a higher frailty index [36], and Luo et al. showed that frailty increased the incidence of exacerbations within 1 year in COPD patients [37]. Previous research also demonstrated that frailty raised the risk of all‐cause mortality in COPD patients [37, 38]. Although in this study, the frailty group had a higher all‐cause mortality rate, there was no statistical difference compared with the robust group. Earlier studies followed patients for a longer period [37, 38], while this study only had a 1‐year follow‐up, which may explain the finding. Taken together, these results indicate that frailty was an important comorbidity that can contribute to poor outcomes in COPD patients.

COPD is a heterogeneous condition, and it is difficult to evaluate it using a single index. Frailty, as a major comorbidity of COPD, can offer useful information on prognosis. Through our analysis, frailty was in similar predictive value to dyspnoea and the exacerbation history for future exacerbation and could further augment the predictive abilities of dyspnoea and the exacerbation history for future exacerbation. It's notable that the combination of frailty and dyspnoea performed similarly with the exacerbation history in predictive value of future exacerbation. Also, we compared BODE index with the combination of frailty and dyspnoea as well as the combination of frailty and exacerbation history and the data revealed that frailty combined with either dyspnoea or the exacerbation history exhibited comparable predictive efficacy for future COPD exacerbations to BODE index (Tables S1 and S2). Dyspnoea was believed to be the most common symptom of COPD [16], with simple assessment using mMRC, and patients could generally and accurately describe the severity of current dyspnoea. Although the exacerbation history was often the single strongest predictor for future exacerbation [19], patients may lack the clinical knowledge to judge exacerbations accurately, which may lead to incorrect reporting. Previous work has raised this concern [21]. Neither dyspnoea nor the exacerbation history could provide comprehensive and accurate predictive information alone in COPD patients [15]. In this study, the frailty assessment encompasses five dimensions: weight loss, exhaustion, physical activity, walking time, and grip strength [9], which could reflect the function of various human organ systems, and unlike the exercise capacity component of the BODE index [22], this frailty assessment is easy to implement at the outpatient department. Considering the above results and findings, we emphasize the importance of frailty as the multi‐dimensional assessment for COPD patients apart from symptoms and the exacerbation history, and that frailty could be incorporated into a new index in combination with dyspnoea or the exacerbation history. Another suggestion is that, due to the more objectiveness of frailty and dyspnoea than the exacerbation history, the combination of frailty and dyspnoea may serve as useful supplementary information when patients cannot provide an accurate exacerbation count.

In this study, the AUC of all relative factors was modest (about 0.60–0.65). This study was conducted in real‐world conditions, and many confounding factors may be involved. Although adjustments were made for baseline characteristics, five comorbidities, and treatment adherence, there may still be unrecognized comorbidities, unreported exacerbations, or other influencing factors.

Regarding the application of frailty assessment in COPD management, factors independently associated with frailty can serve as screening indicators to initially identify COPD patients at high risk of frailty. As a well‐documented risk factor for future exacerbation, frailty has been verified to augment the predictive value of dyspnoea and exacerbation history for subsequent exacerbation. This suggests that frailty is capable of stratifying high‐risk COPD patients and could optimize the prognostic risk classification system for COPD. Bronchodilator and inhaled corticosteroids were fundamental treatments for COPD patients [15]. A previous study has shown that interventions such as exercise, nutritional supplementation and cognitive training can reverse frailty in older adults [39]. Maddocks et al. found that pulmonary rehabilitation could reduce mMRC scores, improve health status and reverse frailty in COPD patients [40]. Future work should explore how to incorporate frailty with inhaled therapies or physical interventions in COPD management.

This study has several limitations. First, 88.6% of participants were male. In our previous work and in Chinese national data, males represent the majority of COPD patients, and they also have higher exposure to smoking [24, 30], which may explain this distribution. These findings should be assessed in a larger cohort with more female COPD patients. Second, we assessed only the grip strength for muscle function, but further detections including dual‐energy X‐ray absorptiometry or multifrequency bioelectrical impedance analysis were not performed to diagnose sarcopenia. There should be more investigations between sarcopenia and frailty in the future. Third, this was a single‐centre study with a relatively small sample size, so the results should be examined in larger, multicentre cohorts and validated externally. Fourth, the follow‐up period should be extended to obtain more comprehensive information on all‐cause mortality.

## 5 Conclusion

Elderly patients with COPD experience a high prevalence of frailty, and those with frailty are characterized by older age, lower BMI, poorer educational attainment, more severe symptom burden, and worse pulmonary function. Frailty, as a crucial comorbidity in COPD, contributes to an elevated risk of future exacerbation, and integrating frailty with dyspnoea or the exacerbation history yields an improved predictive value for future exacerbation, which indicates that frailty could be considered seriously as a comprehensive and multidimensional assessment in the clinical management of COPD.

## Funding

This work was supported by the National Natural Science Foundation of China (NSFC, Grant 81970044) and a Xiangya Mingyi grant (2013).

## Ethics Statement

This study (RealDTC study, the analysis of current status in diagnosis and treatment of COPD, ChiCTR‐POC‐17010431) was approved by the local ethics committee of the Xiangya Second Hospital and conducted in accordance with the Declaration of Helsinki (2016076). Written informed consent was obtained from all the participants.

## Conflicts of Interest

The authors declare no conflicts of interests.

## Supporting information


**Table S1:** The AUC of the BODE index for future exacerbation, frequent exacerbation and hospitalization by ROC curve.
**Table S2:** The Delong test was used to compare the ability of the ROC curve of the BODE index and risk index (1 and 2) for future exacerbation, frequent exacerbation and hospitalization.

## Data Availability

All data of this study are available from the corresponding author, Ping Chen, upon reasonable request.

## References

[1] S. A. Christenson , B. M. Smith , M. Bafadhel , and N. Putcha , “Chronic Obstructive Pulmonary Disease,” Lancet 399 (2022): 2227–2242, 10.1016/s0140-6736(22)00470-6. 35533707

[2] P. Hanlon , X. Guo , E. McGhee , J. Lewsey , D. McAllister , and F. S. Mair , “Systematic Review and Meta‐Analysis of Prevalence, Trajectories, and Clinical Outcomes for Frailty in COPD,” npj Primary Care Respiratory Medicine 33 (2023): 1, 10.1038/s41533-022-00324-5. PMC9816100 36604427

[3] N. Tzanakis , G. Hillas , F. Perlikos , et al., “Managing Comorbidities in COPD,” International Journal of Chronic Obstructive Pulmonary Disease 10 (2015): 95–109, 10.2147/copd.S54473. PMC4293292 25609943

[4] C. Wang , X. Guo , X. Xu , et al., “Association Between Sarcopenia and Frailty in Elderly Patients With Chronic Kidney Disease,” Journal of Cachexia, Sarcopenia and Muscle 14 (2023): 1855–1864, 10.1002/jcsm.13275. PMC10401549 37300354

[5] R. M. Collard , H. Boter , R. A. Schoevers , et al., “Prevalence of Frailty in Community‐Dwelling Older Persons: A Systematic Review,” Journal of the American Geriatrics Society 60 (2012): 1487–1492, 10.1111/j.1532-5415.2012.04054.x. 22881367

[6] Y. Gao , H. Zhang , K. Fang , et al., “The Relationship Between Frailty, BMI, and Mortality in Older Adults: Results From the CLHLS,” BMC Geriatrics 25 (2025): 539, 10.1186/s12877-025-06197-w. PMC12275446 40684122

[7] X. Mu , X. Wang , Q. Zeng , et al., “Sarcopenia and the Frailty Progression Among Chinese: A Longitudinal Study,” Frontiers in Public Health 13 (2026): 1551282, 10.3389/fpubh.2025.1551282. PMC12812598 41561850

[8] H. Arai , K. Maeda , H. Wakabayashi , et al., “Diagnosis and Outcomes of Cachexia in Asia: Working Consensus Report From the Asian Working Group for Cachexia,” Journal of Cachexia, Sarcopenia and Muscle 14 (2023): 1949–1958, 10.1002/jcsm.13323. PMC10570088 37667992

[9] L. P. Fried , C. M. Tangen , J. Walston , et al., “Frailty in Older Adults: Evidence for a Phenotype,” Journals of Gerontology. Series A, Biological Sciences and Medical Sciences 56, no. 3 (2001): M146–M156, 10.1093/gerona/56.3.m146. 11253156

[10] F. Petermann‐Rocha , J. P. Pell , C. Celis‐Morales , and F. K. Ho , “Frailty, Sarcopenia, Cachexia and Malnutrition as Comorbid Conditions and Their Associations With Mortality: A Prospective Study From UK Biobank,” Journal of Public Health (Oxford, England) 44 (2022): e172–e180, 10.1093/pubmed/fdaa226. PMC9234318 33423060

[11] P. Hanlon , B. I. Nicholl , B. D. Jani , D. Lee , R. McQueenie , and F. S. Mair , “Frailty and Pre‐Frailty in Middle‐Aged and Older Adults and Its Association With Multimorbidity and Mortality: A Prospective Analysis of 493 737 UK Biobank Participants,” Lancet Public Health 3 (2018): e323–e332, 10.1016/s2468-2667(18)30091-4. PMC6028743 29908859

[12] L. de Souza Dias , A. C. Galvão Ferreira , J. L. Rodrigues da Silva Junior , et al., “Prevalence of Frailty and Evaluation of Associated Variables Among COPD Patients,” International Journal of Chronic Obstructive Pulmonary Disease 15 (2020): 1349–1356, 10.2147/copd.S250299. PMC7297564 32606644

[13] S. Kagiali , D. Inal‐Ince , A. Cakmak , et al., “Daily Living Activities, Exercise Capacity, Cognition, and Balance in COPD Patients With and Without Frailty,” Irish Journal of Medical Science 191 (2021): 817–824, 10.1007/s11845-021-02654-8. 34028643

[14] K. Ushida , A. Shimizu , S. Hori , Y. Yamamoto , and R. Momosaki , “Hospital Frailty Risk Score Predicts Outcomes in Chronic Obstructive Pulmonary Disease Exacerbations,” Archives of Gerontology and Geriatrics 100 (2022): 104658, 10.1016/j.archger.2022.104658. 35190332

[15] M. Cazzola , J. Bajpai , L. Calzetta , M. G. Matera , and P. Rogliani , “GOLD 2026: Transforming COPD Management With Early Intervention, Multi‐Dimensional Assessment, and Personalized Care,” Drugs 86 (2026): 581–597, 10.1007/s40265-026-02303-3. PMC13109179 41806208

[16] C. D. Blinderman , P. Homel , J. Andrew Billings , J. A. Billings , S. Tennstedt , and R. K. Portenoy , “Symptom Distress and Quality of Life in Patients With Advanced Chronic Obstructive Pulmonary Disease,” Journal of Pain and Symptom Management 38 (2009): 115–123, 10.1016/j.jpainsymman.2008.07.006. 19232893

[17] C. Casanova , J. M. Marin , C. Martinez‐Gonzalez , et al., “Differential Effect of Modified Medical Research Council Dyspnea, COPD Assessment Test, and Clinical COPD Questionnaire for Symptoms Evaluation Within the New GOLD Staging and Mortality in COPD,” Chest 148 (2015): 159–168, 10.1378/chest.14-2449. 25612228

[18] Q. Song , L. Lin , W. Cheng , et al., “Clinical–Functional Characteristics and Risk of Exacerbation and Mortality Among More Symptomatic Patients With Chronic Obstructive Pulmonary Disease: A Retrospective Cohort Study,” BMJ Open 13, no. 3 (2023): e065625, 10.1136/bmjopen-2022-065625. PMC10032416 36944469

[19] J. R. Hurst , J. Vestbo , A. Anzueto , et al., “Susceptibility to Exacerbation in Chronic Obstructive Pulmonary Disease,” New England Journal of Medicine 363, no. 12 (2010): 1128–1138, 10.1056/NEJMoa0909883. 20843247

[20] J. J. Soler‐Cataluna , “Severe Acute Exacerbations and Mortality in Patients With Chronic Obstructive Pulmonary Disease,” Thorax 60 (2005): 925–931, 10.1136/thx.2005.040527. PMC1747235 16055622

[21] D. M. G. Halpin , H. Healey , D. Skinner , V. Carter , R. Pullen , and D. Price , “Exacerbation History and Blood Eosinophil Count Prior to Diagnosis of COPD and Risk of Subsequent Exacerbations,” European Respiratory Journal 64 (2024): 2302240, 10.1183/13993003.02240-2023. PMC11447287 39147410

[22] B. R. Celli , C. G. Cote , J. M. Marin , et al., “The Body‐Mass Index, Airflow Obstruction, Dyspnea, and Exercise Capacity Index in Chronic Obstructive Pulmonary Disease,” New England Journal of Medicine 350, no. 10 (2004): 1005–1012, 10.1056/NEJMoa021322. 14999112

[23] J. M. Marin , S. J. Carrizo , C. Casanova , et al., “Prediction of Risk of COPD Exacerbations by the BODE Index,” Respiratory Medicine 103 (2009): 373–378, 10.1016/j.rmed.2008.10.004. 19013781

[24] Q. Song , C. Liu , W. Cheng , et al., “Clinical Characteristics and Risk of All‐Cause Mortality in Low Education Patients With Chronic Obstructive Pulmonary Disease in the Chinese Population,” Journal of Global Health 13 (2023): 05163, 10.7189/jogh.13.04163. PMC10693353 38033249

[25] Y.‐y. Zhao , C. Liu , Y.‐Q. Zeng , et al., “Modified and Simplified Clinically Important Deterioration: Multidimensional Indices of Short‐Term Disease Trajectory to Predict Future Exacerbations in Patients With Chronic Obstructive Pulmonary Disease,” Therapeutic Advances in Respiratory Disease 14 (2020): 1753466620977376, 10.1177/1753466620977376. PMC7768878 33357117

[26] J.‐x. Duan , W. Cheng , Y.‐q. Zeng , et al., “Characteristics of Patients with Chronic Obstructive Pulmonary Disease Exposed to Different Environmental Risk Factors: A Large Cross‐Sectional Study,” International Journal of Chronic Obstructive Pulmonary Disease 15 (2020): 2857–2867, 10.2147/copd.S267114. PMC7654530 33192059

[27] D. Zhao , Y. Zhou , C. Jiang , Z. Zhao , F. He , and P. Ran , “Small Airway Disease: A Different Phenotype of Early Stage COPD Associated With Biomass Smoke Exposure,” Respirology 23 (2017): 198–205, 10.1111/resp.13176. 28906034

[28] Y. Li , F. Wen , Q. Ma , et al., “Use of CAPTURE to Identify Individuals Who May or May Not Require Treatment for Chronic Obstructive Pulmonary Disease,” American Journal of Respiratory and Critical Care Medicine 208 (2023): 435–441, 10.1164/rccm.202303-0504OC. 37315325

[29] O. Le Rouzic , N. Roche , A. B. Cortot , et al., “Defining the “Frequent Exacerbator” Phenotype in COPD,” Chest 153 (2018): 1106–1115, 10.1016/j.chest.2017.10.009. 29054347

[30] C. Wang , J. Xu , L. Yang , et al., “Prevalence and Risk Factors of Chronic Obstructive Pulmonary Disease in China (The China Pulmonary Health [CPH] Study): A National Cross‐Sectional Study,” Lancet 391 (2018): 1706–1717, 10.1016/s0140-6736(18)30841-9. 29650248

[31] L.‐C. Yan , H.‐Y. Lu , X.‐Y. Wang , et al., “Prevalence and Risk Factors of Frailty in Patients With Chronic Obstructive Pulmonary Disease: Systematic Review and Meta‐Analysis,” European Geriatric Medicine 14 (2023): 789–802, 10.1007/s41999-023-00800-2. PMC10447286 37436687

[32] P.‐J. Chen , K.‐Y. Yang , W.‐C. Perng , et al., “Effect of Dyspnea on Frailty Stages and Related Factors in Taiwanese Men With COPD,” International Journal of Chronic Obstructive Pulmonary Disease 13 (2018): 2463–2469, 10.2147/copd.S172694. PMC6101740 30147312

[33] L. Yuan , M. Chang , and J. Wang , “Abdominal Obesity, Body Mass Index and the Risk of Frailty in Community‐Dwelling Older Adults: A Systematic Review and Meta‐Analysis,” Age and Ageing 50 (2021): 1118–1128, 10.1093/ageing/afab039. 33693472

[34] N. Putcha , A. R. Anzueto , P. M. A. Calverley , et al., “Mortality and Exacerbation Risk by Body Mass Index in Patients With COPD in TIOSPIR and UPLIFT,” Annals of the American Thoracic Society 19 (2022): 204–213, 10.1513/AnnalsATS.202006-722OC. PMC8867355 34406915

[35] R. Zhou , G. Tian , X. Guo , and R. Li , “Lung Function and the Risk of Frailty in the European Population: A Mendelian Randomization Study,” European Journal of Medical Research 29 (2024): 95, 10.1186/s40001-024-01685-y. PMC10832278 38297347

[36] S. Scarlata , P. Finamore , A. Laudisio , et al., “Association Between Frailty Index, Lung Function, and Major Clinical Determinants in Chronic Obstructive Pulmonary Disease,” Aging Clinical and Experimental Research 33 (2021): 2165–2173, 10.1007/s40520-021-01878-z. 34009526

[37] J. Luo , D. Zhang , W. Tang , et al., “Impact of Frailty on the Risk of Exacerbations and All‐Cause Mortality in Elderly Patients With Stable Chronic Obstructive Pulmonary Disease,” Clinical Interventions in Aging 16 (2021): 593–601, 10.2147/cia.S303852. PMC8053481 33880018

[38] S. Y. Lee , M. S. Z. Nyunt , Q. Gao , et al., “Co‐Occurrence of Physical Frailty and COPD and Association With Disability and Mortality,” Chest 161 (2022): 1225–1238, 10.1016/j.chest.2021.12.633. 34914976

[39] T. P. Ng , L. Feng , M. S. Z. Nyunt , et al., “Nutritional, Physical, Cognitive, and Combination Interventions and Frailty Reversal Among Older Adults: A Randomized Controlled Trial,” American Journal of Medicine 128 (2015): 1225–1236.e1, 10.1016/j.amjmed.2015.06.017. 26159634

[40] M. Maddocks , S. S. C. Kon , J. L. Canavan , et al., “Physical Frailty and Pulmonary Rehabilitation in COPD: A Prospective Cohort Study,” Thorax 71 (2016): 988–995, 10.1136/thoraxjnl-2016-208460. PMC5099190 27293209

