# Supplementary material for: The Clinical Characteristic and Prognostic Value of Frailty in Elderly COPD Patients
Source: J Cachexia Sarcopenia Muscle. 2026 Sep 24;17(5):e70381. doi: 10.1002/jcsm.70381 (PMC13612742; doi:10.1002/jcsm.70381)
Supplement: Supplementary file 1 [file JCSM-17-e70381-s001.docx]

Supplementary table 1 The AUC of BODE index for future exacerbation, frequent exacerbation and hospitalization by ROC curve.

| **Factors** | **Any exacerbation** | | **Frequent exacerbation** | | **Hospitalization** | |
| --- | --- | --- | --- | --- | --- | --- |
|  | **AUC (95%CI)** | **P value** | **AUC (95%CI)** | **P value** | **AUC (95%CI)** | **P value** |
| **BODE index** | 0.621 (0.570-0.671) | <0.001 | 0.612 (0.556-0.668) | <0.001 | 0.617 (0.562-0.672) | <0.001 |

Note: BODE index were defined as: (1). Body Mass Index (B), 0 equals BMI >21 kg/m², 1 equals BMI ≤21 kg/m²;

(2). Airflow Obstruction (O, FEV₁ % predicted), 0 equal ≥65%, 1 equals 50%–64%, 2 equals 36%–49%, 3 equals ≤35%;

(3). Dyspnea (D, mMRC grade), 0 equals Grade 0–1; 1 equals Grade 2; 2 equals Grade 3; 3 equals Grade 4;

(4). Exercise Capacity (E, 6-minute walk distance), 0 equals ≥ 350 m, 1 equals 250–349 m, 2 equals 150–249 m, 3 equals ≤ 149 m.

Risk Stratification of BODE Index: Score 0–2: Low risk, Score 3–4: Moderate risk, Score 5–6: High risk, Score 7–10: Very high risk.

Supplementary table 2 The Delong test was used to compare the ability of ROC curve of BODE index and risk index (1 and 2) for future exacerbation, frequent exacerbation and hospitalization.

| **Compared Factors** | **Any exacerbation** | | **Frequent exacerbation** | | **Hospitalization** | |
| --- | --- | --- | --- | --- | --- | --- |
|  | **Difference of AUC** | **P value** | **Difference of AUC** | **P value** | **Difference of AUC** | **P value** |
| **Risk index 1 vs BODE index** | -0.018 | 0.373 | 0.007 | 0.742 | 0.001 | 0.968 |
| **Risk index 2 vs BODE index** | 0.015 | 0.509 | 0.037 | 0.150 | 0.006 | 0.830 |

Note: We defined that robust was 0, pre-frailty was 1, frailty was 2, less dyspnea was 0, more dyspnea was 1, no exacerbation history was 0 and one or more exacerbation history was 1. Risk index 1 was the combination of frailty and dyspnea, and Risk index 2 was the combination of frailty and the exacerbation history.

BODE index were defined as: (1). Body Mass Index (B), 0 equals BMI >21 kg/m², 1 equals BMI ≤21 kg/m²;

(2). Airflow Obstruction (O, FEV₁ % predicted), 0 equal ≥65%, 1 equals 50%–64%, 2 equals 36%–49%, 3 equals ≤35%;

(3). Dyspnea (D, mMRC grade), 0 equals Grade 0–1; 1 equals Grade 2; 2 equals Grade 3; 3 equals Grade 4;

(4). Exercise Capacity (E, 6-minute walk distance), 0 equals ≥ 350 m, 1 equals 250–349 m, 2 equals 150–249 m, 3 equals ≤ 149 m.

Risk Stratification of BODE Index: Score 0–2: Low risk, Score 3–4: Moderate risk, Score 5–6: High risk, Score 7–10: Very high risk.
